# Maize *Unstable factor for orange1* Is Required for Maintaining Silencing Associated with Paramutation at the *pericarp color1* and *booster1* Loci

**DOI:** 10.1371/journal.pgen.1002980

**Published:** 2012-10-04

**Authors:** Rajandeep S. Sekhon, Po-Hao Wang, Lyudmila Sidorenko, Vicki L. Chandler, Surinder Chopra

**Affiliations:** 1 Department of Plant Science, Pennsylvania State University, University Park, Pennsylvania, United States of America; 2 Plant Biology Graduate Program, Pennsylvania State University, University Park, Pennsylvania, United States of America; 3 BIO5 Institute and Department of Plant Sciences, University of Arizona, Tucson, Arizona, United States of America; The University of North Carolina at Chapel Hill, United States of America

## Abstract

To understand the molecular mechanisms underlying paramutation, we examined the role of *Unstable factor for orange1* (*Ufo1*) in maintaining paramutation at the maize *pericarp color1* (*p1*) and *booster1* (*b1*) loci. Genetic tests revealed that the *Ufo1-1* mutation disrupted silencing associated with paramutation at both *p1* and *b1*. The level of up regulation achieved at *b1* was lower than that at *p1*, suggesting differences in the role *Ufo1-1* plays at these loci. We characterized the interaction of *Ufo1-1* with two silenced *p1* epialleles, *P1-rr*′ and *P1-pr^TP^*, that were derived from a common *P1-rr* ancestor. Both alleles are phenotypically indistinguishable, but differ in their paramutagenic activity; *P1-rr′* is paramutagenic to *P1-rr*, while *P1-pr^TP^* is non-paramutagenic. Analysis of cytosine methylation revealed striking differences within an enhancer fragment that is required for paramutation; *P1-rr*′ exhibited increased methylation at symmetric (CG and CHG) and asymmetric (CHH) sites, while *P1-pr^TP^* was methylated only at symmetric sites. Both silenced alleles had higher levels of dimethylation of lysine 9 on histone 3 (H3K9me2), an epigenetic mark of silent chromatin, in the enhancer region. Both epialleles were reactivated in the *Ufo1-1* background; however, reactivation of *P1-rr′* was associated with dramatic loss of symmetric and asymmetric cytosine methylation in the enhancer, while methylation of up-regulated *P1-pr^TP^* was not affected. Interestingly, *Ufo1-1*–mediated reactivation of both alleles was accompanied with loss of H3K9me2 mark from the enhancer region. Therefore, while earlier studies have shown correlation between H3K9me2 and DNA methylation, our study shows that these two epigenetic marks are uncoupled in the *Ufo1-1*–reactivated *p1* alleles. Furthermore, while CHH methylation at the enhancer region appears to be the major distinguishing mark between paramutagenic and non-paramutagenic *p1* alleles, H3K9me2 mark appears to be important for maintaining epigenetic silencing.

## Author Summary

Natural allelic variability is crucial for genetic improvement. While the genetic mechanisms leading to such variation have been studied in depth, relatively less is known about the role of epigenetic mechanisms in generation of allelic diversity. Paramutation is a phenomenon in which one allele can silence another allele in *trans* and, once established, such epigenetic silencing is heritable. To further understand the molecular components of paramutation, we characterized two epialleles of the *pericarp color1* (*p1*) gene of maize, which originated from a common progenitor; however, only one of these alleles is paramutagenic. Results show that, while both alleles have high levels of symmetric (CG and CHG) methylation in a distal enhancer element, only the paramutagenic allele has higher levels of asymmetric (CHH) methylation. Since CHH methylation is imposed and maintained through RNA–mediated mechanisms, these results indicate that paramutation at the *p1* locus involves RNA–mediated silencing pathway. Further, both silent epialleles are reactivated in the presence of an unlinked dominant mutation *Ufo1-1*, and reactivation is accompanied by the loss of suppressive histone mark H3K9me2. Finally, we show that *ufo1* is also required for epigenetic silencing at the *booster1* locus and thus affects additional loci in maize that participate in paramutation.

## Introduction

Paramutation, originally described at the *r1* (*red1*) locus in maize [1], refers to the exchange of epigenetic information between two alleles in a heterozygote that leads to heritable change in expression of one of the alleles. In maize, regulatory genes involved in the synthesis of flavonoids -anthocyanins and phlobaphenes- have been extensively used to study paramutation. Thus far, paramutation has been described for four loci involved in flavonoid biosynthesis: *r1* (*red1*) and *b1* (*booster1*) encode basic helix-loop-helix (bHLH) transcription factors while *pl1* (*purple plant1*) and *p1* (*pericarp color1*) encode R2R3 *Myb* transcription factors [2]. Of these *r1*, *b1*, and *pl1* regulate biosynthesis of anthocyanins while *p1* regulates biosynthesis of phlobaphenes. Paramutation-like phenomena have also been reported for another gene in maize [3], and other plants and animals [4], [5].

Detailed characterization of paramutation at the *b1* locus demonstrated that seven 853-bp tandem repeats located ∼100-kb upstream of the transcription start site are required for paramutation [6], [7]. Cloning of *mediator of paramutation1* (*mop1*) revealed that an RNA-dependent RNA polymerase (RDR), most similar to Arabidopsis RDR2, is required for establishment and maintenance of paramutation [8] and indicated that RNA-mediated chromatin silencing regulates paramutation [9]. The role of RNA-mediated silencing mechanisms in paramutation has been further strengthened by the cloning of two additional maize genes. Of these, *required to maintain repression6* (*rmr6*) encodes largest subunit of RNA polymerase IV (Pol IV) similar to Arabidopsis NRPD1 [10] and *Mop2/rmr7* encodes second-largest subunit similar to Arabidopsis NRPD2/E2 [11], [12] which functions in both Pol IV and Pol V complexes [13]. Pol IV and Pol V are plant-specific polymerases which function in the biogenesis of small RNAs and in the RNA-mediated chromatin silencing pathway [13]–[15].

The *p1* locus has been used as a model system to understand molecular and epigenetic mechanisms that regulate tissue-specific gene expression. *p1*-controlled red phlobaphene pigments accumulate in pericarp, cob glumes, husk, silk and tassel glumes. Alleles of *p1* are identified based on a two-letter suffix, which denotes their expression in the pericarp and cob glume, respectively [16], [17]. Variable pigmentation patterns of several *p1* alleles have been attributed not only to DNA sequence differences, but also to differential epigenetic states [18]–[21]. For instance, *P1-rr* and *P1-wr*, two prototype *p1* alleles have distinct phenotypes; *P1-rr* produces red pericarp and red cob glume while *P1-wr* specifies white pericarp and red cob glume phenotypes. These alleles have major structural differences: *P1-rr* is a single copy gene while *P1-wr* is composed of six or more gene copies tandemely arranged in a head-to-tail fashion [18]. Both alleles have similar coding and regulatory sequences and functional analyses have identified similar basal promoter and proximal enhancer regions [22], [23]. However, *P1-rr* has fully pigmented pericarp whereas *P1-wr* accumulates no pigment in this tissue. The difference in pericarp expression pattern has been attributed to higher DNA methylation within the regulatory sequences of *P1-wr* in comparison to *P1-rr* which displays very low levels of DNA methylation [18]. Several *p1* alleles that show a *P1-rr*-like pericarp and cob glume pigmentation phenotype and carry a *P1-wr* mutlicopy gene structure have been shown to be hypomethylated [21]. Likewise, loss of pericarp and cob glume pigmentation of P1-wr*, a silent epiallele of *P1-wr*, has been attributed to even denser cytosine methylation than that of *P1-wr* [20].

Presence of *Unstable factor for orange1* (*Ufo1*), an un-cloned *trans*-acting dominant modifier, induces loss of cytosine methylation from *P1-wr* and P1-wr*, thereby relieving epigenetic suppression and leading to ectopic gain of phlobaphene pigmentation in various plant organs including dried silk, tassel glume, husk, and leaf sheath [20], [24], [25]. *Ufo1*-induced phenotypes show incomplete penetrance (some progeny carrying the mutation completely lack the mutant phenotype) and poor expressivity (the extent of mutant phenotype is variable).

Paramutation at the *p1* locus was observed due to the interactions of the endogenous *P1-rr* allele with a transgene carrying fragments of the *P1-rr* regulatory region. The transgene was composed of a 1.2-kb *P1-rr* distal enhancer fragment (P1.2) located 5-kb upstream of *P1-rr* transcription start site, a basal (b) *p1* promoter fragment (−236 to +326 untranslated leader), GUS coding region, and *PinII* terminator [26]. When plants with this transgene (P1.2b::GUS) were crossed with those carrying the *P1-rr* allele, a subset of the transgenic progeny showed a striking reduction in pericarp and cob glume pigmentation. The silenced state of *P1-rr*, designated as *P1-rr*′, was inherited independent of the transgene, and it could silence the naïve *P1-rr* allele. Importantly, the silenced, paramutagenic state of *P1-rr*′ was associated with increased DNA methylation within the P1.2 fragment that is required for paramutation [26]. Additional transgenic experiments revealed that the P1.2 fragment is required and sufficient for paramutation [27]. The *mop1-1* and *Mop2-1* mutations disrupt paramutation of *P1-rr* to *P1-rr′* demonstrating that RNA mediated mechanisms are involved in establishment of silencing associated with *p1* paramutation [11], [27]. Maintenance of silencing is less dependent on RNA mediated mechanisms, as up to three consecutive generations of exposure to the *mop1-1* mutation were required for *P1-rr′* up regulation while *Mop2-1* had no effect on *P1-rr′* silencing even after three generations of continuous exposure [11].

To further elucidate the mechanism(s) underlying epigenetic regulation of paramutation, we characterized involvement of *Ufo1* in the regulation of silencing associated with *b1* and *p1* paramutation. We compared effects of the *Ufo1-1* mutation on paramutagenicity and densities of DNA methylation within the P1.2 fragment in *P1-rr′* and another spontaneous epimutation of *P1-rr*, *P1-pr^TP^* (patterned pericarp and red cob glume). This study highlights the role of histone modifications and DNA methylation and relationship between origin, epigenetic state and differential paramutagenic behavior of these epialleles derived from the common progenitor *P1-rr* allele. Possible mechanisms dictating different effects of *Ufo1-1* on paramutation at *b1* and *p1* loci are discussed.

## Results

### *Ufo1* reactivates the silent *P1-rr′* allele

**Figure 1:**
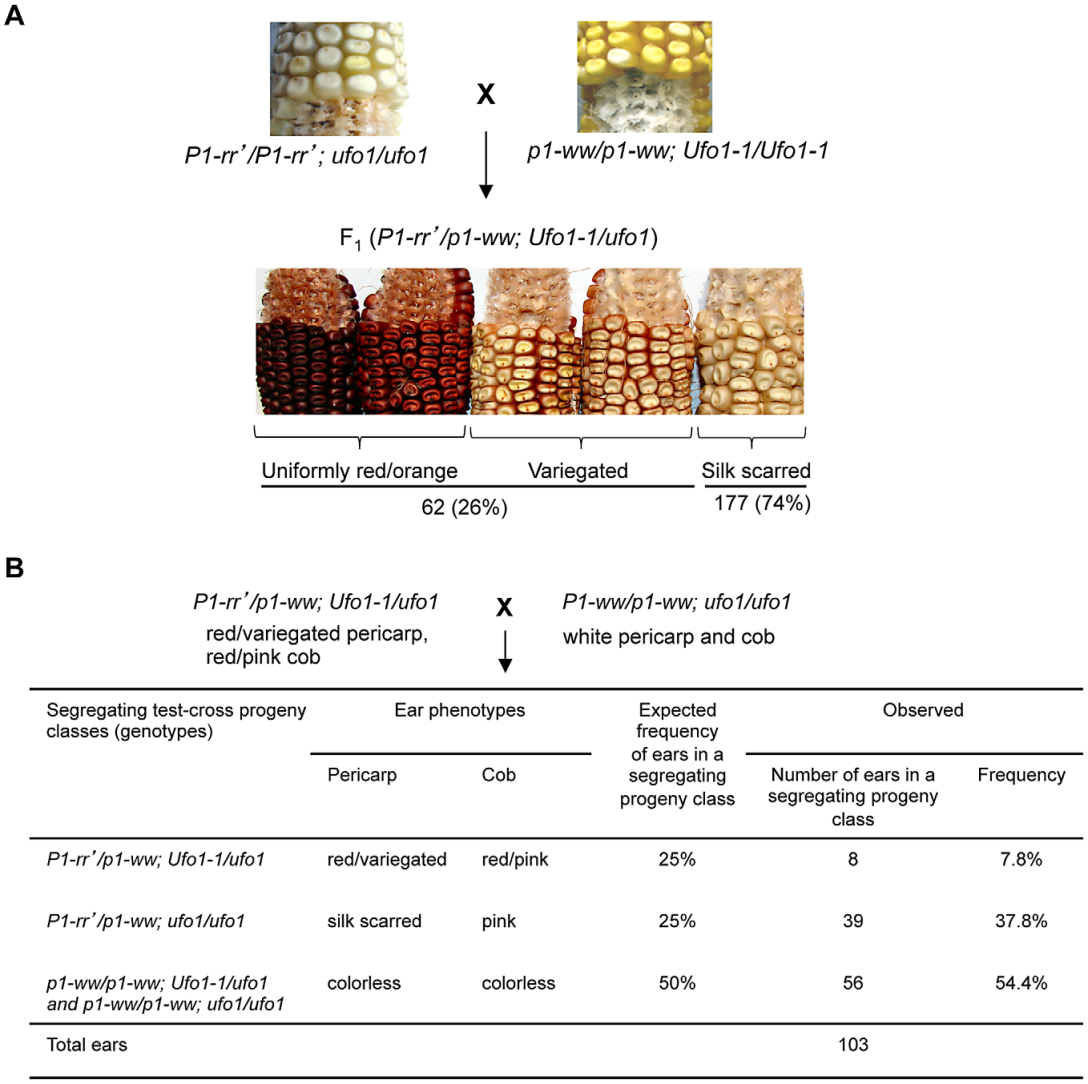
*Ufo1-1* mediates reactivation of *P1-rr′*. A. Pericarp and cob glume phenotypes of F_1_ progeny plants obtained from a cross between highly suppressed *P1-rr′ ufo1* and *p1-ww Ufo1-1*. B. Heritability of *Ufo1-1*-induced reactivation of *P1-rr′*. F_1_ plants were crossed with *p1-ww*[*4Co63*] and progeny was scored for pericarp pigmentation. Expected segregation frequencies are based on the assumption that *P1-rr′* reverts back to silenced state after segregation of *Ufo1-1*.

To test if *Ufo1-1* reactivates single-copy silenced *p1* alleles, *P1-rr′ ufo1* plants were crossed with *p1-ww Ufo1-1* (Figure 1A). Of 239 F_1_ plants screened, 62 (26%) showed gain of pericarp pigmentation while 177 (74%) remained silent as indicated by a colorless pericarp except red pigmentation at the point where silk attaches to pericarp during seed development (hereafter referred to as silk scarred phenotype). Similar to earlier published results [20], [24], the effect of *Ufo1-1* on *P1-rr′* silencing was not fully penetrant as only a subset of the F_1_ progeny showed gain of pigmentation with pericarp phenotypes varying from uniformly red or orange to red/orange variegation of pericarps. To test the heritability of reactivated *P1-rr′* phenotypes, F_1_ plants showing gain of pericarp pigmentation were crossed with *p1-ww*[*4Co63*] (Figure 1B). As expected, approximately half of the progeny (54.4%) had colorless pericarp and cob glume specified by the homozygosity for the recessive *p1-ww* allele ears (χ^2^ = 0.79; P = 0.38). If *Ufo1-1* mediated *P1-rr′* up regulation were not heritable then 25% of total progeny is expected to carry *Ufo1-1* and *P1-rr′* and have up regulated pericarp and cob glume pigmentation phenotype, while 25% of progeny carrying *P1-rr′* and a wild type *ufo1* allele should have silenced silk scarred ears. Results of the analysis demonstrated that 37.8% of ears had silk scarred *P1-rr′* phenotype and 7.8% had up regulated red or orange pericarp and cob glume pigmentation phenotypes. This segregation ratio indicates that the reactivated *P1-rr′* state is not heritable and reverts back to silenced state after segregation of *Ufo1-1*.

**Figure 2:**
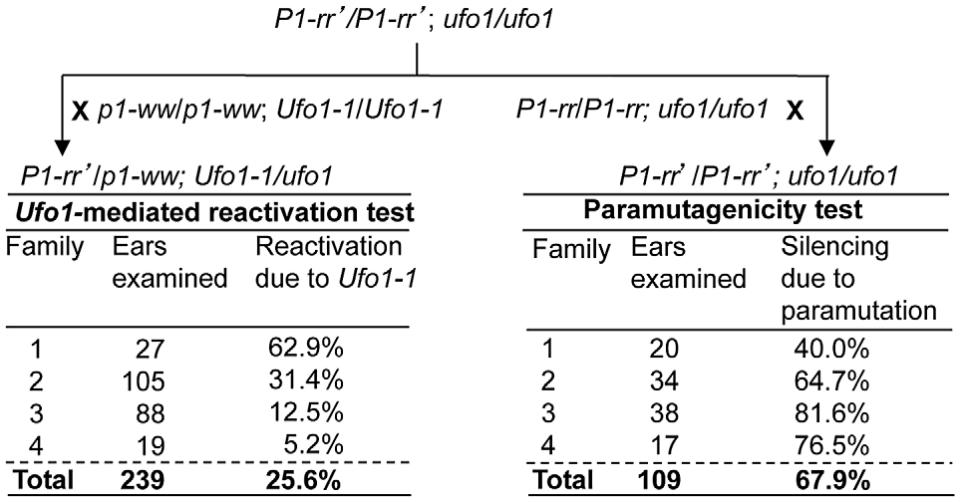
Paramutagenicity of *P1-rr′* inversely correlates with frequency of reactivation by *Ufo1-1*. Crossing scheme is shown at the top. Tables show results of tests for *P1-rr′* paramutagenicity (on the right) and *Ufo1-1* mediated *P1-rr′* reactivation (on the left) for the same *P1-rr′* plants.

The paramutagenic activity of individual *P1-rr′* families is highly variable and the suppression of naive *P1-rr* by *P1-rr′* can range from 0 to >90% [26]. We tested if *P1-rr′* families with differential paramutagenic activity also differ for their extent of activation by *Ufo1*. Representative *P1-rr*′ plants were crossed with *p1-ww Ufo1-1* and each *P1-rr′* plant was also crossed with the paramutable naïve *P1-rr* allele (Figure 2). Scoring the resultant progeny for frequency of paramutation revealed that *P1-rr′* paramutagenicity varied between 40 and 81.6%, while reactivation by *Ufo1-1* varied between 5.2% and 62.9%. Interestingly, frequency of reactivation by *Ufo1-1* was lower in the progeny of the highly paramutagenic *P1-rr*′ (families 3 and 4) as compared to the progeny of the low paramutagenic *P1-rr*′ (families 1 and 2). Statistical analysis revealed that high paramutagenic ability negatively correlated with frequency of reactivation by *Ufo1-1* (R^2^ = −0.86). This result demonstrates that the mechanism(s) governing the paramutagenic activity also interfere with the effect of the *Ufo1-1* mutation on *P1-rr′*.

### *Ufo1-1*–induced reactivation is associated with hypomethylation of *P1-rr′*

**Figure 3:**
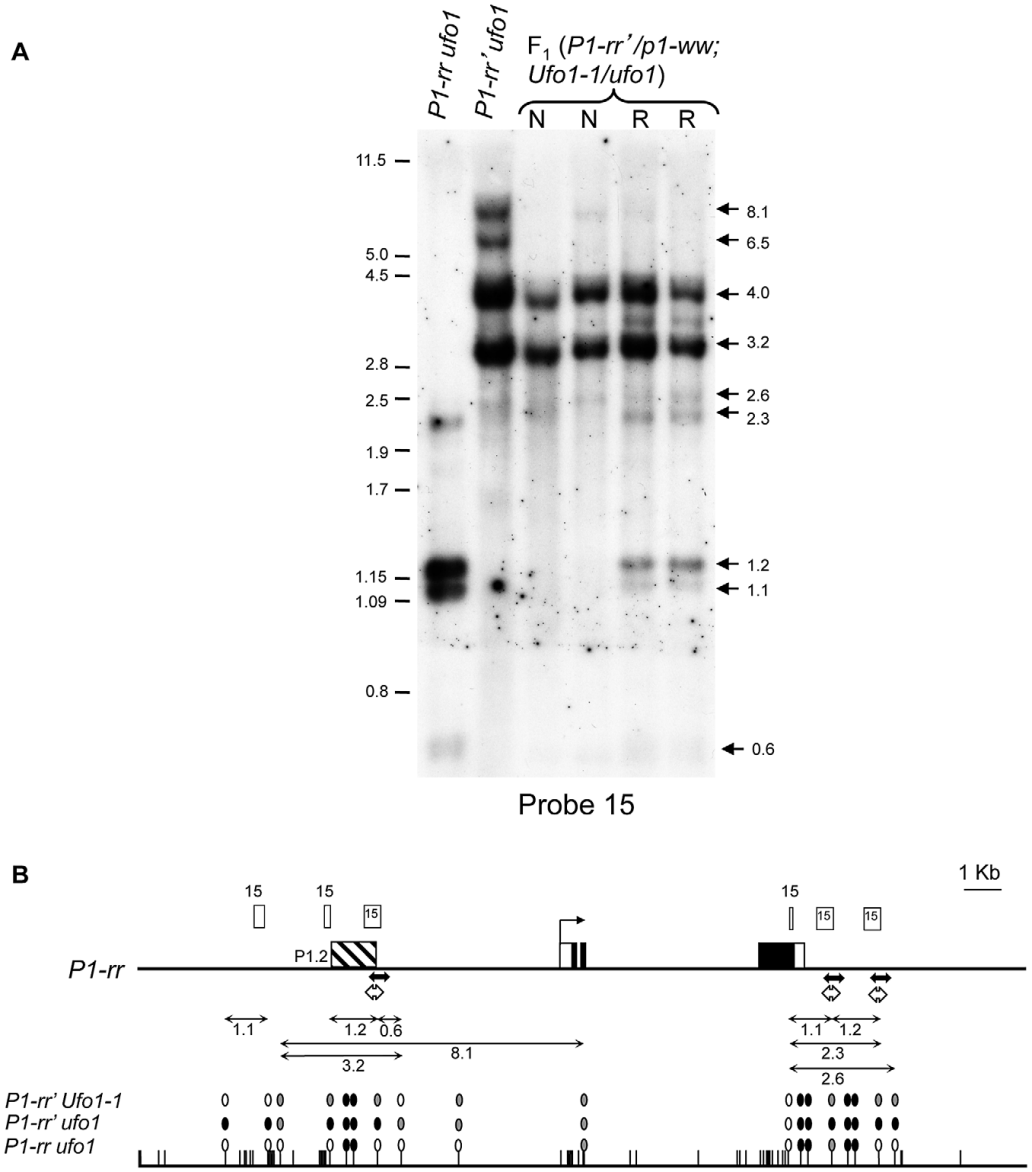
*Ufo1-1*-mediated loss of cytosine methylation in *P1-rr*′. A. DNA gel blot showing methylation differences among *P1-rr ufo1*, *P1-rr*′*ufo1*, two F_1_ sibling plants not reactivated by *Ufo1-1* (marked as N), and two F_1_ sibling plants reactivated by *Ufo1-1* (marked as R). Leaf genomic DNA was digested with *Hpa*II endonuclease and hybridized with probe fragment 15. Molecular weight marker in kilobase pair is shown on the left, and arrows on the right show positions and sizes of the hybridizing bands. B. Methylation map of *P1-rr ufo1*, *P1-rr′ ufo1* and *P1-rr′ Ufo1-1*. Gene structure is shown at the top. A solid black line represents *P1-rr* sequence with black boxes denoting exons and open boxes indicating UTR's. Transcription start site is indicated as a bent arrow. Positions and relative sizes of the probe 15 fragments are shown as open boxes above the gene map; three relatively smaller boxes labeled as 15 are the truncated copies of probe 15. Striped box to the left of transcription start site represents the P1.2 enhancer fragment. Solid double-headed arrows below the gene map indicate the region examined by genomic bisulfite sequencing while hollow double-headed arrows represent the region analyzed by ChIP assay. Methylation map of *Hpa*II sites is shown below the gene structure. Solid line represents the *P1-rr* sequence while the short vertical lines represent *Hpa*II sites. Ovals above the *Hpa*II sites represent methylation status of individual sites; open, grey and black ovals represent non-methylated, partially methylated, and completely methylated *Hpa*II sites, respectively. Lines with double arrowheads above the methylation map represent the *Hpa*II fragments observed on the DNA gel blot; only fragments and *Hpa*II sites resolved by the restriction analysis are shown.

The silenced state of *P1-rr′* is characterized by increased methylation of the P1.2 enhancer element [26]. To test if the reactivation of *P1-rr*′ by *Ufo1-1* involved hypomethylation within the P1.2 enhancer sequence, *p1* fragment 15 was used as a probe to hybridize gel blots carrying leaf genomic DNA digested with methylation sensitive endonuclease *Hpa*II (Figure 3) and with a combination of *Hpa*II and methylation insensitive endonuclease *Dra*I (data not shown). In *P1-rr*, two major fragments of approximately 1.2 and 1.1-kb are observed (Figure 3A) which originate from unmethylated *Hpa*II sites flanking the fragment 15 in the upstream promoter and downstream of 3′ untranslated region (UTR) (Figure 3B). Most of these sites are methylated in *P1-rr*′ resulting in the loss of 1.2 and 1.1-kb bands and appearance of higher molecular weight bands indicating increased DNA methylation at these *Hpa*II sites (Figure 3B). Examination of restriction patterns revealed loss of DNA methylation in all reactivated (R) and non-activated (N) *P1-rr′/p1-ww*; *Ufo1-1/+* plants, as seen from the loss of high (8.1 and 6.5-kb) molecular weight bands. However, additional loss of DNA methylation from *Hpa*II sites that flank fragment 15 are observed in the R plants which results in the reappearance of 1.2 and 1.1-kb bands. This result demonstrates that partial loss of DNA methylation occurs in all reactivated *P1-rr′/p1-ww; Ufo1-1/+* plants, but greater hypomethylation is observed in plants with up regulated pericarp and cob glume pigmentation phenotype.

**Figure 4:**
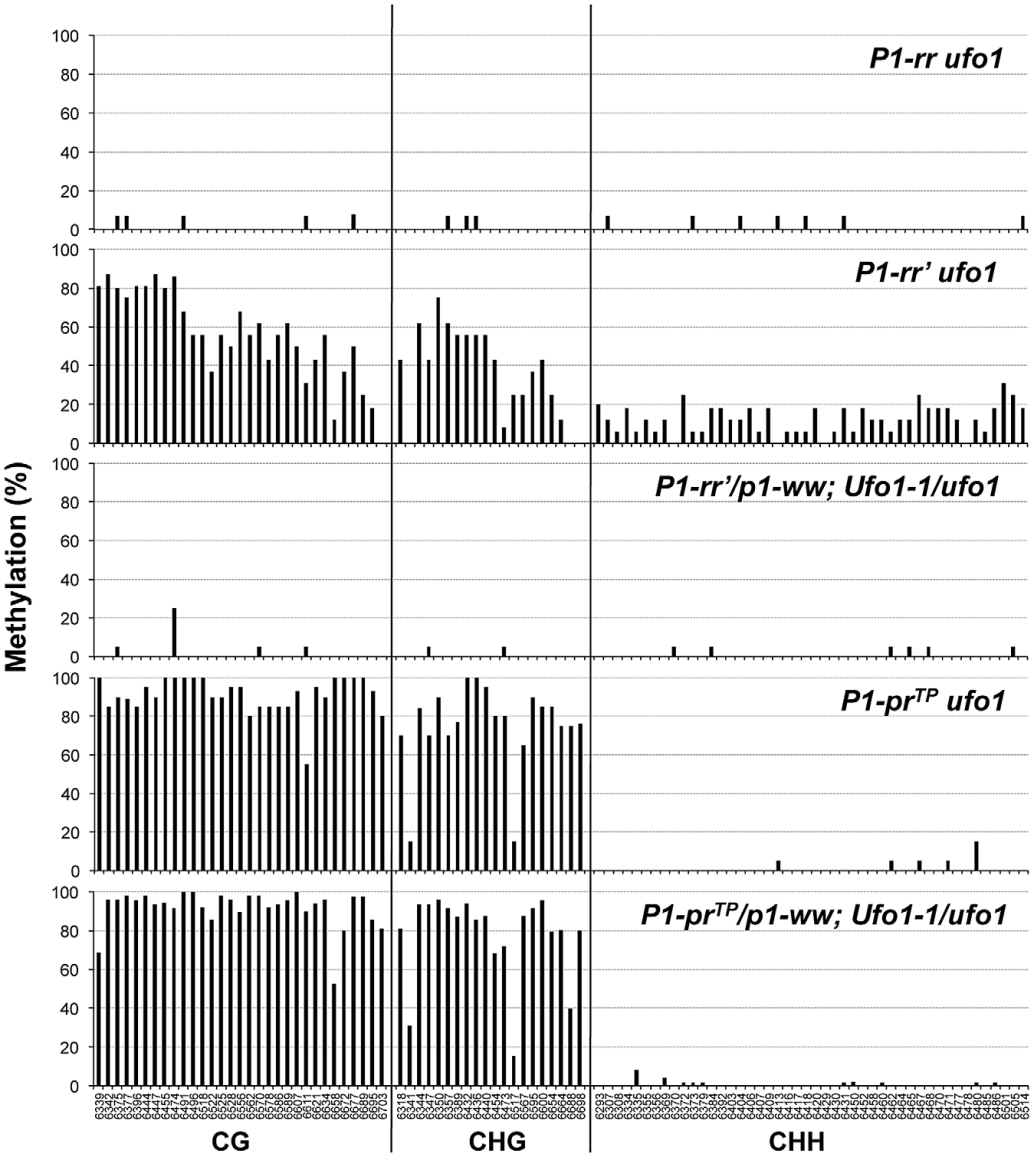
*Ufo1-1*-induced methylation modifications of individual cytosine residues in the 443-bp fragment of the P1.2 enhancer of *P1-rr′* and *P1-pr^TP^*. *P1-rr′/p1-ww; Ufo1-1/ufo1* and *P1-pr^TP^/p1-ww; Ufo1-1/ufo1* refer to plants that showed gain of pigmentation. Bisulfite sequencing was performed on genomic DNA extracted from pericarp tissue. Genomic DNA from two independent plants per genotype was used for bisulfite sequencing. For each genotype, the percent methylation is shown on the *y*-axis while the position of cytosine residues in CG, CHG, and CHH context is shown at the *x*-axis of the bottom graph.

To assay methylation of individual cytosine residues, we performed genomic bisulfite sequencing on the upper strand of a 443-bp fragment of the P1.2 enhancer region, which is required for paramutation. Since *P1-rr* is expressed in pericarp and *Ufo1*-*1* induced gain of pigmentation is more pronounced in this tissue, we used pericarp DNA for the methylation assay. In the functional *P1-rr* allele, almost all of the symmetric (CG and CHG; H is A, T, or C), and asymmetric (CHH) cytosine sites were un-methylated (Figure 4). Silencing of *P1-rr*′ was associated with hypermethylation of most symmetric sites, and to a lesser extent, with that of asymmetric sites (Figure 4, Figure S1). Additionally, symmetric cytosine methylation was higher in the 5′ end and it was reduced toward the 3′ end of the P1.2 fragment. Analysis of reactivated *P1-rr*′*Ufo1-1* plants revealed that DNA methylation was dramatically reduced at all symmetric and asymmetric cytosines to a level comparable with that of the *P1-rr ufo1* plants. Therefore, the DNA gel blot and bisulfite sequencing analyses demonstrated that *Ufo1-1*-mediated reactivation of *P1-rr′* correlates with reduction of methylation within the P1.2 enhancer fragment.

### *P1-pr^TP^* is a silenced, hypermethylated epiallele of *P1-rr*

**Figure 5:**
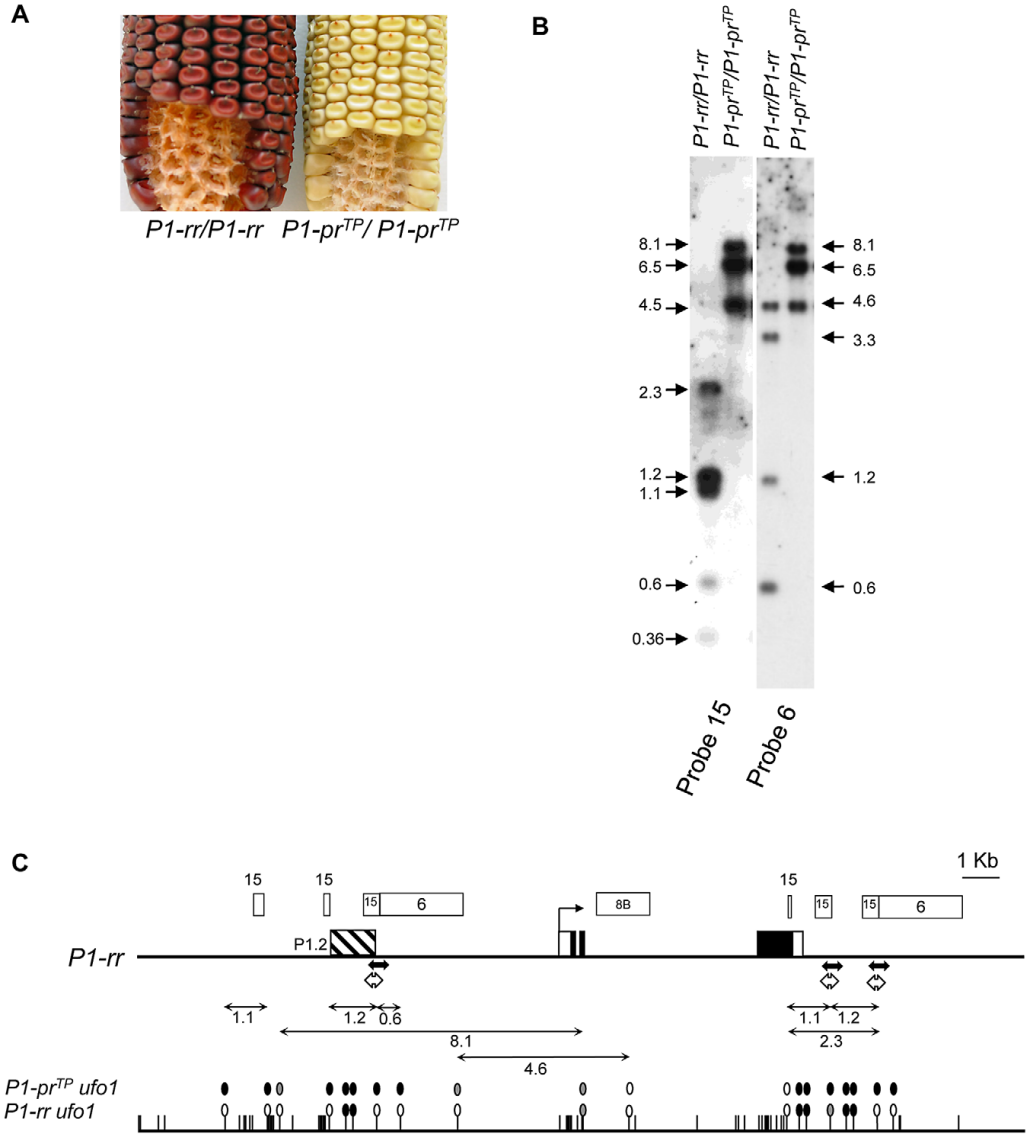
Silencing of *P1-pr^TP^*, an epiallele of *P1-rr*, is correlated with DNA methylation. A. Loss of pericarp and cob glume pigmentation phenotype of *P1-pr^TP^*. The pericarp pigmentation is present only at the silk attachment point while the cob glumes are pink. B. *P1-pr^TP^* is highly methylated as compared to *P1-rr*. Leaf genomic DNA of *P1-rr* and *P1-pr^TP^* was digested with *Hpa*II and sequentially hybridized with *p1* probe fragments 15 and 6 (See Figure 5C for location of probes). Size and location of hybridizing bands in kilobase pair is indicated by arrows on the left for probe 15 and on the right for probe 6. C. Methylation map of *P1-rr ufo1* and *P1-pr^TP^ ufo1*. Gene structure is shown at the top and the rest of the figure description is same as in Figure 3B.

In an earlier study, Das and Messing [19] described a *P1-pr* (patterned pericarp and red cob glume) epiallele of *P1-rr* with reduced pericarp pigmentation. *P1-pr* silencing correlated with increased DNA methylation and failure of the P1.2 enhancer [28] to undergo tissue-specific chromatin remodeling in pericarp [19], [29]. We characterized another independently isolated spontaneous epiallele of *P1-rr, P1-pr^TP^*, which is phenotypically similar to *P1-pr* [19] and *P1-rr′* [26] with colorless or silk scarred pericarp and light pink to light red cob glume (Figure 5A). The silenced *P1-pr^TP^* state is very stable as out of ∼1,000 plants screened, none showed any spontaneous gain of pericarp pigmentation (data not shown).

Extensive DNA gel blot analysis and partial sequencing revealed that the *P1-pr^TP^* sequence is identical to *P1-rr* (not shown). To test if *P1-pr^TP^* silencing is associated with epigenetic modification, we compared DNA methylation of *Hpa*II-digested *P1-pr^TP^* and *P1-rr*. In *P1-rr*, the probe fragment 15 hybridized to five restriction fragments including the 1.2 kb and 1.1 kb bands (Figure 5B). Loss of these bands and appearance of higher molecular weight bands in *P1-pr^TP^* showed that the region around the P1.2 enhancer was methylated (Figure 5C). Similarly, upon hybridization with the probe fragment 6, *P1-pr^TP^* produced higher molecular weight bands as compared to *P1-rr* indicating methylation within the promoter region. However, *P1-pr^TP^* and *P1-rr* had similar methylation levels in the coding region as shown by hybridization with probe fragment 8B (Figure S2). Taken together, these results demonstrate that *P1-pr^TP^* is an epiallele of *P1-rr* and that silencing of *P1-pr^TP^* is associated with DNA methylation of upstream regulatory regions including the P1.2 enhancer.

### *P1-pr^TP^* is not paramutagenic to *P1-rr*

To test if *P1-pr^TP^* participates in paramutation, it was crossed with *P1-rr* and resulting F_1_ progenies were scored for pericarp pigmentation. All 300 F_1_ plants screened showed red pericarp and red cob glume phenotype indicating that *P1-pr^TP^* is non-paramutagenic to *P1-rr* and behaves as a recessive allele. Moreover, in the F_2_ generation, *P1-rr* and *P1-pr^TP^* phenotypes segregated in the expected 3∶1 ratio further supporting that *P1-pr^TP^* does not participate in paramutation (data not shown). To summarize, genetic data indicated that *P1-pr^TP^* is not paramutagenic to a naive *P1-rr*.

### *Ufo1-1* disrupts the suppressed state of *P1-pr^TP^*

**Figure 6:**
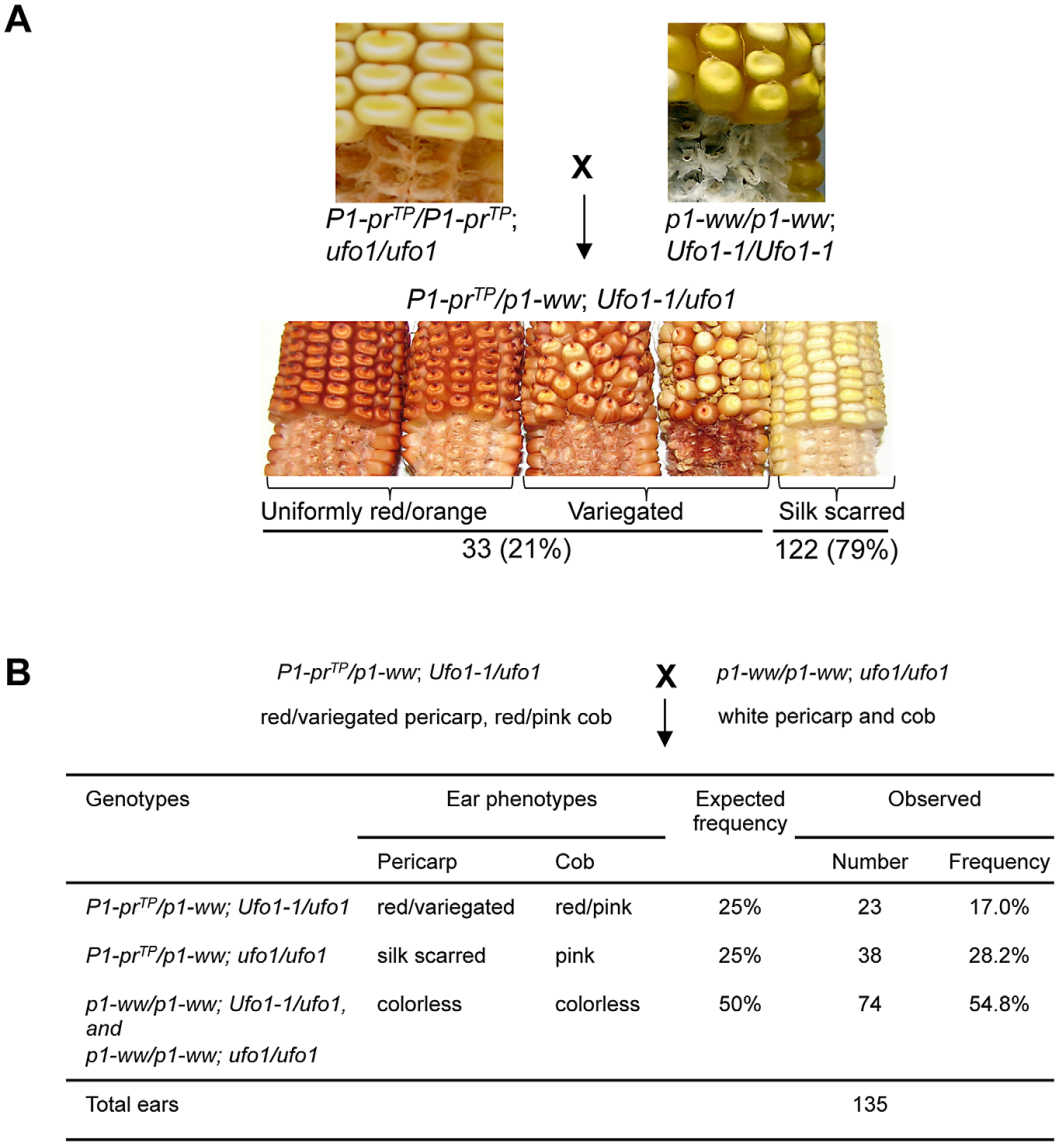
Representative data showing *Ufo1-1*–induced reactivation of *P1-pr^TP^*. A. Pericarp and cob glume pigmentation phenotypes of F_1_ progeny obtained from a cross between *P1-pr^TP^* and *p1-ww Ufo1-1* plants. B. Heritability of *Ufo1-1*-induced reactivation of *P1-pr^TP^*. F_1_ plants showing gain of pericarp pigmentation (red/variegated pericarps) were crossed with *p1-ww*[*4Co63*] and test-cross progenies were examined for pericarp and cob glume pigmentation. Expected segregation frequencies are based on assumption that increased pigmentation of *P1-pr^TP^* allele is not heritable in the absence *Ufo1-1*.

To test if the *Ufo1-1* mutation can disrupt the silenced state of *P1-pr^TP^*, genetic crosses were performed (Materials and Methods, Figure 6A). Analysis of 155 F_1_ plants revealed that *P1-pr^TP^* was upregulated by the *Ufo1-1* mutation and, similar to *P1-rr′*, frequency of up regulation was low as only 21% of plants exhibited gain in pericarp and cob glume pigmentation (Figure 6A). Further, the F_2_ progeny of *Ufo1-1* expressing plants did not produce the expected phenotypic ratio of 9∶3∶3∶1 (data not shown). To assay whether *Ufo1-1*-mediated reactivation of *P1-pr^TP^* was heritable, F_1_ plants with an increased pericarp and cob glume pigmentation were crossed with colorless *p1-ww*[*4Co63*] plants carrying a wild type *ufo1* allele (Figure 6B). As expected, 50% of the progeny was homozygous for *p1-ww* and therefore produced colorless and pericarp and cob glume (χ^2^ = 1.25; P = 0.263). If the activated state of *P1-pr^TP^* returned to a silenced state after segregation of *Ufo1-1*, 25% of progeny was expected to have a silenced phenotype, while the remaining 25%, still carrying *Ufo1-1*, would have increased pigmentation. There were 28.2% of silk scarred and 17% of red/variegated pericarp and cob glume individuals indicating that, similar to *P1-rr′*, *P1-pr^TP^* up regulation was not heritable in the absence of *Ufo1-1*. Overall, these observations demonstrate that *Ufo1-1* temporarily disrupts the silenced epigenetic state of *P1-pr^TP^* leading to increased pericarp pigmentation. Non-heritable *P1-pr^TP^* gain of pigmentation in the mutant *Ufo1-1* background is reminiscent of *Ufo1-1* interaction with other *p1* alleles [20], [24].

### *Ufo1-1*–induced reactivation of *P1-pr^TP^* occurs in the presence of DNA methylation within the P1.2 enhancer

Gel blot analysis involving *Hpa*II-digested leaf genomic DNA hybridized with *p1* probe fragments did not detect any methylation differences between the silenced and reactivated *P1-pr^TP^* plants (Figure S2). We also performed genomic bisulfite sequencing of the 443 bp of the P1.2 enhancer in pericarp of *P1-pr^TP^ ufo1* and *P1-pr^TP^/p1-ww; Ufo1-1/ufo1* plants. In comparison to *P1-rr′*, which is hypermethylated in symmetric and asymmetric contexts, *P1-pr^TP^* is hypermethylated only in symmetric contexts (Figure 4). No decrease in DNA methylation was observed in pericarps of *P1-pr^TP^/p1-ww* plants that were strongly up regulated by *Ufo1-1*. These results are in striking contrast with *P1-rr′* where *Ufo1-1*-up regulation correlated with dramatic reduction of cytosine methylation and demonstrates that similar phenotypes of *P1-rr′* and *P1-pr^TP^* are specified by distinct molecular mechanisms.

### *Ufo1-1*–induced reactivation is associated with depletion of suppressive histone marks on *P1-rr′* and *P1-pr^TP^*

**Figure 7:**
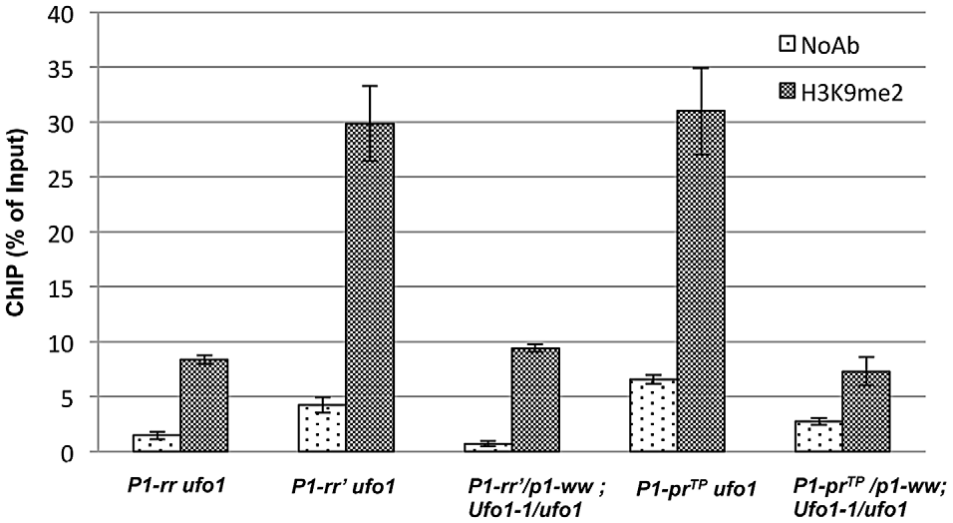
Comparison of H3K9-dimethylation (H3K9me2) levels in *P1-rr′* and *P1-pr^TP^* in the absence and presence of *Ufo1-1*. ChIP assay was performed using pericarp tissues. Chromatin complex was immunoprecipitated with antibodies against H3K9me2. Mouse IgG was used as a negative control (NoAb). Quantitative PCR was performed to quantify the DNA enrichments at the P1.2 kb distal enhancer. The data presented here is the mean ± SE from three biological replicates of three independent ChIP experiments.

Histone 3 lysine 9 dimethylation (H3K9me2) is a histone modification associated with heterochromatin assembly and transcriptional silencing. To test if histone modifications are involved in the silencing of *P1-rr′* and *P1-pr^TP^*, chromatin immunoprecipitation (ChIP) followed by quantitative real-time PCR (qPCR) was performed to determine the H3K9me2 enrichments at the P1.2 region. The ChIP assays showed that the chromatin encompassing the 1.2 kb distal enhancer is significantly enriched for H3K9me2 in *P1-rr′* and *P1-pr^TP^* plants as compared to *P1-rr* (Figure 7). Thus, irrespective of their involvement in paramutation, silenced state of both alleles is associated with enrichment of the suppressive H3K9me2 mark.

To further investigate if *ufo1* is involved in maintenance of H3K9me2, ChIP assays were performed at *P1-rr′* and *P1-pr^TP^* in the presence of *Ufo1-1*. Interestingly, in the presence of *Ufo1-1*, there is a dramatic reduction of H3K9me2 within the 1.2 kb distal enhancer region of *P1-rr′* and *P1-pr^TP^* (Figure 7). To summarize, these results demonstrate that *ufo1* plays a role in maintaining repressive H3K9me2 histone marks at *P1-rr′* and *P1-pr^TP^*.

### *Ufo1-1* disrupts silencing of the paramutated *b1* allele

**Figure 8:**
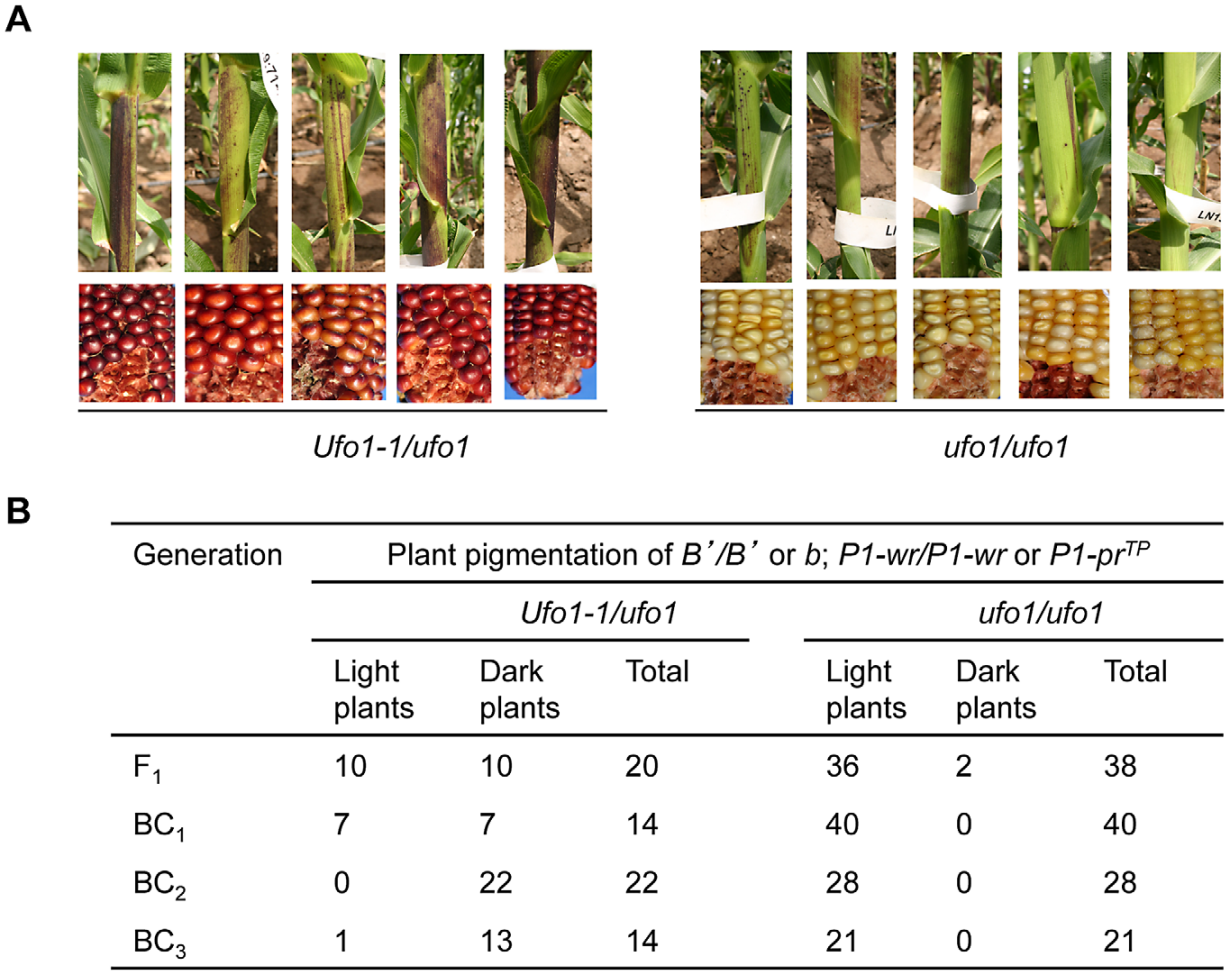
*Ufo1-1* increases *B′* pigmentation. Effect of *Ufo1-1* was assayed in four consecutive backcrosses as shown in the crossing scheme in Figure S3. A. Photo panels from BC_2_ generation showing *B′* plant pigmentation and corresponding *P1-wr* pericarp and cob glume phenotypes, which were used as phenotypic indicators of *Ufo1-1* presence. The *P1-wr* has colorless pericarp and red cob and *P1-pr^TP^* has silk-scarred pericarp and pink cob glume in the *ufo1* genetic background. In the mutant *Ufo1-1/ufo1* background *P1-wr* and *P1-pr^TP^* exhibit dramatic increase in pigmentation and have very dark red pericarp and cob glume. In the wild type *ufo1* background, the *B′* allele has light streaky plant pigmentation while in the *Ufo1-1/ufo1* background *B′* plants have increased pigmentation as evidenced by the presence of broad darkly pigmented sectors. This increase of *B′* pigmentation is significant because *B′* never displays any increase in pigmentation in wild type genetic backgrounds. B. Summary of the results from F_1_ and three generations of introgression in the mutant *Ufo1-1/ufo1* background are shown.

Paramutation at the *b1* locus occurs when the highly expressing, darkly pigmented *B-I* allele is exposed to a low expressing, lightly pigmented *B′* allele in heterozygote [4]. While paramutable *B-I* is unstable and spontaneously reverts to *B′* at variable frequencies, paramutagenic *B′* is stable in wild type genetic backgrounds. To test if *Ufo1*-*1*-mediated disruption of epigenetic silencing associated with paramutation extends beyond the *p1* locus, the silenced *B′* allele was introgressed into the *Ufo1-1* background and *B′*-specified plant pigmentation was evaluated (Figure S3). Examination of the segregating progeny revealed that *B′*-specified pigmentation increased in the mutant (*Ufo1-1/ufo1*), as evident from multiple, wide and darkly pigmented sectors on sheaths (Figure 8A), husks, and tassels (not shown). Extent of *B′* pigmentation in the *Ufo1-1* background was moderate and never reached solid dark purple plant pigment observed in *B-I* or dark pigmentation phenotypes observed for *B′* in *mop1-1* [8], [9] and *mop2-1* [11] mutant backgrounds. The *Ufo1-1* mutation was penetrant in about half of F_1_ plants, and this number, but not intensity of *B′* pigmentation, increased in later backcrosses (BC_2_ and BC_3_) (Figure 8B). Overall, these results show that the *Ufo1-1* mutation disrupts the silenced state of *B′* allele and therefore *ufo1* is involved in the pathway that regulates silencing associated with paramutation at multiple loci.

## Discussion

This study provides further insights into the mechanisms underlying epigenetic silencing and paramutation in maize. Despite sharing a common *P1-rr* progenitor allele, the two epialleles described here are distinct in that *P1-rr′* is paramutagenic while *P1-pr^TP^* is neutral and does not participate in paramutation. Silencing of both these alleles is associated with increased cytosine methylation within the P1.2 distal enhancer. Hypermethylation in *P1-rr′* is observed in all cytosine contexts while the methylation in *P1-pr^TP^* is restricted to CG and CHG contexts. Both alleles, however, have enrichment in H3K9me2 in this region suggesting that this suppressive mark is involved in maintenance of silencing in both alleles. Presence of *Ufo1-1* alleviates epigenetic suppression which is associated with dramatic reduction of H3K9me2. We also show here that *Ufo1-1* disrupts the paramutation-associated silencing of the *B′* allele. Thus *ufo1* is involved in the maintenance of epigenetic silencing originating spontaneously (epimutation), and that during paramutation.

### Unique cytosine methylation patterns of *P1-rr′* and *P1-pr^TP^* indicate involvement of distinct mechanisms in silencing of these alleles

Given the fact that *P1-rr′* and *P1-pr^TP^* are epialleles of a common progenitor allele (*P1-rr*), their differential paramutagenic ability is intriguing; while *P1-rr′* is able to communicate its chromatin state *in trans* to *P1-rr*, *P1-pr^TP^* fails to do so. A notable difference between these epialleles is the nature of their origin: *P1-rr′* silencing was induced by a transgene carrying P1.2 distal enhancer sequence, while *P1-pr^TP^* originated spontaneously and causal factors for its origin are unknown. Establishment of transgene-mediated silencing at *P1-rr′* requires RNA-dependent RNA polymerase activity of *mop1* [27] and plant-specific RNA polymerase IV/V activity as evidenced by requirement for *mop2* [11] implicating RNA mediated mechanism in the origin of this epiallele. The extent and distribution of cytosine methylation in the distal enhancer region of *P1-rr′* and *P1-pr^TP^* are strikingly different. In *P1-rr′*, this region is methylated in all sequence contexts (CG, CHG, and CHH), and CG and CHG methylation levels are higher in the 5′ and decrease toward the 3′ end, while CHH methylation is evenly distributed throughout the assayed region. These high and low methylation regions correspond, respectively, to the middle and the end of the sequence homology between the endogenous P1.2 enhancer and the transgene fragment that caused the initial silencing. This pattern of symmetric cytosine methylation and increased levels of asymmetric CHH methylation further points to the role of RNA-mediated mechanisms in *P1-rr′* silencing. In *P1-pr^TP^*, however, only CG and CHG sites are methylated and the methylation is uniformly high throughout the assayed region. Furthermore, while *P1-rr′* can, on rare occasions, show spontaneous gain of function [26], silenced state of *P1-pr^TP^* is very stable. While it is possible that initial events leading to the silencing of *P1-pr^TP^* involved RNA mediated mechanism, based on a lack of asymmetric DNA methylation, RNA signals do not appear to contribute to the maintenance of DNA methylation. In summary, difference in the levels of CHH methylation, a hallmark of RdDM, seems to be the only epigenetic mark that correlates with differential paramutagenic ability of the two alleles.

### Loss of suppressive histone mark but not cytosine methylation correlates with *Ufo1-1*–mediated reactivation of silenced alleles

While the paramutagenic (*P1-rr′*) and non-paramutagenic (*P1-pr^TP^*) alleles differ for their cytosine methylation patterns within the P1.2 enhancer, both display enrichment in H3K9me2. Furthermore, while symmetric methylation persists at the enhancer in *Ufo1*-reactivated *P1-pr^TP^*, levels of H3K9me2 are decreased in both reactivated alleles. Thus, H3K9me2 appears to be an indispensable repressive epigenetic mark for maintaining silencing at the paramutagenic and non-paramutagenic *p1* epialleles. However, we cannot rule out that, both H3K9me2 and DNA methylation play important and mutually reinforcing roles in maintenance of silencing at both *P1-pr^TP^* and *P1-rr′*. In a similar study at the *b1* locus, tandem repeats critical for paramutation exhibited tissue-independent DNA methylation, while enrichment in H3K9me2 and H3K27me2 was tissue-specific [30]. Based on these results, authors concluded that H3K9me2 does not play a role in the mitotic heritability of the silenced *B′* state, but rather serves to reinforce silencing in a tissue-specific manner [30]. In our study, H3K9me2 enrichment was observed in pericarp tissue of *P1-rr′* and *P1-pr^TP^* pericarps, and loss of silencing in *Ufo1-1* correlated with loss of H3K9me2. However, it remains unclear whether this mark is involved in tissue-specific regulation and reinforcement of silencing, or it also plays a role in tissue-independent maintenance of silencing.

Several studies have reported a positive correlation between cytosine, especially CHG, methylation and H3K9me2 marks [31]-[34]. In Arabidopsis, loss of KRYPTONITE, an H3K9-specific methyltransferase, results in a loss of both H3K9me2 and CHG methylation [35] and a genome-wide survey of H3K9me2 and CHG methylation has shown very high correlation between the two epigenetic marks [32]. In our study, CHG methylation and not H3K9me2 persists in the enhancer of reactivated *P1-pr^TP^ Ufo1-1* plants indicating that H3K9me2 and CHG methylation may exist independent of each other. However, our data does not exclude the possibility that CHG methylation is crucial for establishment of silencing in *P1-Pr^TP^*.

### Activation of *P1-rr′* by *Ufo1-1* is inversely correlated with paramutagenicity

The paramutagenic ability of *P1-rr′* is highly variable; silencing of a naive *P1-rr* allele by *P1-rr′* in independent families varied between 0–95% in the current and an earlier study [26]. We demonstrate that the paramutagenic ability of *P1-rr′* was inversely correlated with *Ufo1-1*-induced reactivation. Thus, highly paramutagenic *P1-rr′* stocks interfere with *Ufo1-1*-mediated reactivation in a manner not currently understood. Variable levels of *p1* alleles reactivation have been attributed to incomplete penetrance of *Ufo1-1*, and to the extent of epigenetic silencing of the *p1* allele involved [20], [24]. *Ufo1-1* induces pericarp and cob glume pigmentation in moderately methylated *P1-wr* plants, but only cob glume pigmentation in a highly methylated P1-wr*. Interestingly, repeated back crosses of P1-wr* Ufo1-1 with *p1-ww Ufo1-1* stock eventually leads to a gain of pericarp pigmentation (R. Sekhon, P. Wang and S. Chopra, unpublished data). Thus, the highly silenced epigenetic states (P1-wr* and highly paramutagenic *P1-rr′* families) may not show immediate gain of pericarp pigmentation in the presence of *Ufo1-1* while moderately silenced states (*P1-wr*) can be readily perturbed.

### *ufo1* has a broader role in diverse epigenetic pathways

The *Ufo1-1* mutant allele perturbs the organ-specific expression patterns of the multicopy *p1* alleles *P1-wr* and P1-wr* [20], [24]. These alleles do not participate in paramutation [20], [26]. Our finding that presence of *Ufo1-1* leads to reactivation of the paramutagenic *P1-rr*′ and *B′* alleles indicates that the wild type factor is involved in maintenance of silencing imposed by paramutation. While the absence of RNA-dependent RNA polymerase MOP1 also leads to reactivation of silenced *P1-rr′* and a gain of pericarp pigmentation in the *P1-wr* allele, several generations of absence of MOP1 are required for such activation [27]. In contrast, lack of *ufo1* for one generation is sufficient to abolish silencing at the *p1* locus. On the other hand, lack of *ufo1* leads only to a partial reactivation of *B′* while lack of MOP1 results in immediate disruption of silencing. It thus appears that epigenetic suppression at these two loci is mediated by distinct but overlapping pathways.

Reactivation of the silenced loci in the absence of *ufo1* suggests that it is directly involved in the epigenetic mechanism(s) responsible for the silencing. *Ufo1-1*-mediated reactivation of *P1-rr′* and also in *P1-wr* [20], [24] is associated with the loss of DNA methylation within regulatory regions whereas the de-repression of *P1-pr^TP^* does not seem to involve any methylation modifications at the region tested. These results support our argument [20] that *ufo1* may not be directly involved in establishing and/or maintaining DNA methylation. Given that reactivation of both *P1-rr′* and *P1-pr^TP^* in the *Ufo1-1* background was associated with a loss of H3K9me2 marks, it appears that *ufo1* is involved in maintaining these heterochromatic marks. Future studies to examine the role of *Ufo1-1* in establishment and maintenance of silencing associated with paramutagenic and non-paramutagenic systems, and cloning of the gene will provide insights into *ufo1*-dependent mechanisms in epigenetic regulation of gene expression.

## Materials and Methods

### Genetic stocks

The maize *P1-rr* allele used in this study is derived from the *P1-rr-4B2* genetic stock [36]. Origin of the *P1-rr*′ stock has been previously described [26]. The *P1-rr′* allele used in this study was progeny of a homozygous (*P1-rr′*/*P1-rr′*) plant that showed strong silencing and had colorless pericarp and light pink cob glume. The *P1-pr^TP^*, a spontaneous epiallele of *P1-rr*, has been previously reported [26] and this epiallele is distinct from the previously characterized *P1-pr* allele [19]. A stock carrying *Ufo1-1* has been described previously [24]. The *Ufo1-1* was introgressed into the inbred line *4Co63* (National Seed Storage Laboratory, Fort Collins, CO), which carries a null *p1-ww* allele. Since the *p1-ww Ufo1-1* plants do not produce phlobaphene pigmentation, presence of *Ufo1-1* was tested by crossing individual plants with *P1-wr*[*W23*] [17]. Ectopic gain of pigmentation in pericarp and other organs in the resulting F_1_ progeny confirmed the presence of *Ufo1-1* in the *p1-ww Ufo1-1* stock [24]. The stock carrying *B′* allele of the *b1* gene was obtained from E.H. Coe, Jr. (University of Missouri, Columbia) and this stock carries the *Pl-sr* allele of the *pl* gene that does not impart *b1*-specified pigmentation to the plant body. All plant stocks used in this study carry functional alleles for the structural genes required for anthocyanin and/or phlobaphene biosynthesis.

### DNA gel blot analysis

Genomic DNA was isolated from the fifth or sixth leaf using CTAB method [37]. Genomic DNA was digested to completion using restriction enzymes, reagents, and protocols from Promega (Madison, WI). DNA gel blot was performed as described previously [20]. DNA fragments of *p1* used as probes in DNA gel blot analysis have been described previously [36], [38].

### Genomic bisulfite sequencing

The upper DNA strand of a 443-bp sub-fragment of the P1.2 fragment required for paramutation, was assayed by genomic bisulfite sequencing. Pericarp tissues were collected 18 days after pollination (DAP) from individual plants and genomic DNA was extracted using modified CTAB method [37]. For each genotype, DNA from two plants was subjected to genomic bisulfite sequencing and pooled results from the assay are presented. Eight micrograms of DNA was completely digested with suitable restriction enzymes that cut outside the amplified fragment of interest. The digested DNA was treated with sodium bisulfite following a previously published protocol [39] with modifications [20]. The promoter region was amplified using nested PCR primers [25]. The resulting PCR products were gel purified, cloned using a TOPO TA cloning kit (Invitrogen, Carlsbad, CA), and 20–30 clones/ligation/genotype were sequenced. Due to the complex *P1-rr* locus structure, the region studied by bisulfite sequencing is present in three places (see Figure 3B for details). The sequenced region is also present in *p1-ww*[*4Co63*] albeit with multiple indels and single nucleotide polymorphisms (R.S. Sekhon and S. Chopra, unpublished). These sequence differences were used to omit the clones that originated from the null *p1-ww* allele. Percent methylation at each cytosine residue was calculated by dividing the number of clones methylated for the residue by the total number of clones for that residue for all of the amplified *P1-rr′* and *P1-pr^TP^* regions.

### Chromatin immunoprecipitation assay and quantitative real-time PCR (ChIP-qPCR)

ChIP assays were performed using pericarp tissues following a modified protocol as described previously [40], [41]. Briefly, pericarp tissues were harvested at 18 DAP and cross-linked with 3% formaldehyde. The chromatin complex was then extracted and sheared to a size range of 0.5 to 1 kb fragments using a Bioruptor (Diagenode, Denville, NJ). The anti-H3K9me2 antibody used for ChIP was kindly provided by Dr. Hiroshi Kimura [41]. This antibody was coupled with sheep anti-mouse IgG Dynabeads M-280 (Invitrogen, Grand Island, NY) and incubated with the sheared chromatin. A normal mouse IgG was used as no antibody control (NoAb). The ChIPed DNA was further purified using QIAquick PCR Purification Kit (QIAGEN, Valencia, CA) and quantified with qPCR. The relative enrichment of H3K9me2 modification was normalized to the input DNA loaded in the ChIP reaction as described previously [40]. The primers specific to the P1.2 kb distal enhancer region used in this study are PW_RTF15-2F (5′-GACGTCTCACCGGCTCACA-3′) and PW_RTF15-2R (5′-ATGCAACGCAACGCTTTG-3′). The relative differences between ChIP assay and input sample were determined using the percentage-of-input method (see ChIP analysis; http://www.invitrogen.com/site/us/en/home/Products-and-Services/Applications/RNAi-Epigenetics-and-Gene-Regulation/Chromatin-Remodeling/Chromatin-Immunoprecipitation-ChIP/chip-analysis.html). Data shown in this study are representative result of three independent experiments.

## Supporting Information

Figure S1 Comparison of overall cytosine methylation in the 443 bp fragment of the P1.2 enhancer region as assayed by genomic bisulfite sequencing. For each genotype, overall methylation in each context was calculated by dividing the number of methylated cytosines by the total number of cytosines in the context in all the clones. Context of methylation is on the *x*-axis and percentage of methylation is on the *y*-axis.
(TIF)

Figure S2 Effect of *Ufo1-1* on methylation of *P1-pr^TP^*. Gel blots carrying *Hpa*II-digested leaf genomic DNA of *P1-rr*, *P1-pr^TP^*, and *P1-pr^TP^ Ufo1-1* plants with varying levels of pericarp pigmentation were hybridized with *p1* probes; fragment 15 (A) and 8B (B). Molecular weights (in kilobases) of DNA ladder is shown on the left and sizes of hybridizing bands are shown on the right. Letters on the top of each lane indicate pericarp pigmentation of *P1-pr^TP^*/*p1-ww*; *Ufo1-1/ufo1*: R for up regulated phenotype with pigmented pericarp; N for non-pigmented pericarps.
(TIF)

Figure S3 Crossing scheme used to test effect of *Ufo1-1/ufo1* on *B′*. Homozygous *B′* plants were crossed with the *Ufo1-1/ufo1* stock which carried the neutral to paramutation *b* allele of the *b1* gene. In the presence of *Ufo1-1/ufo1*, the *P1-wr* or *P1-pr^TP^* plants display increased pericarp and cob glume pigmentation which was used to identify the *Ufo1-1/ufo1* plants in segregating families. In cases when a *Ufo1-1/ufo1* plant did not develop ear or ears failed to set seed, *Ufo1-1/ufo1* plants were identified using a combination of phenotypic traits such as reddish/orange plant pigment, small stature, and characteristically bent tassel. To ensure that *Ufo1-1* was passed on to the next generation, only ears with dark red pericarp pigmentation were used for planting. Because *Ufo1-1* homozygotes did not survive in the hot and dry Arizona summer weather, experiment was carried out by repeated backcrossing *Ufo1-1/ufo1* by the *B′ ufo1* stock. Phenotypes of the up regulated *B′* plants and data for all four generations are shown in Figure 8.
(TIF)

## References

[1] Brink RA (1956) A genetic change associated with the *R* locus in maize which is directed and potentially reversible. Genetics 41: 872–890. 17247669 10.1093/genetics/41.6.872 PMC1224369

[2] Chandler VL, Stam M (2004) Chromatin conversations: mechanisms and implications of paramutation. Nature Reviews Genetics 5: 532–544. 10.1038/nrg1378 15211355

[3] Pilu R, Panzeri D, Cassani E, Cerino Badone F, Landoni M, et al (2009) A paramutation phenomenon is involved in the genetics of maize *low phytic acid1-241* (*lpa1-241*) trait. Heredity 102: 236–245. 18781168 10.1038/hdy.2008.96

[4] Chandler VL (2007) Paramutation: from maize to mice. Cell 128: 641–645. 17320501 10.1016/j.cell.2007.02.007

[5] Chandler V, Alleman M (2008) Paramutation: epigenetic instructions passed across generations. Genetics 178: 1839–1844. 18430919 10.1093/genetics/178.4.1839 PMC2323780

[6] Stam M, Belele C, Dorweiler JE, Chandler VL (2002) Differential chromatin structure within a tandem array 100 kb upstream of the maize *b1* locus is associated with paramutation. Genes and Development 16: 1906–1918. 12154122 10.1101/gad.1006702 PMC186425

[7] Stam M, Belele C, Ramakrishna W, Dorweiler JE, Bennetzen JL, et al (2002) The regulatory regions required for *B′* paramutation and expression are located far upstream of the maize *b1* transcribed sequences. Genetics 162: 917–930. 12399399 10.1093/genetics/162.2.917 PMC1462281

[8] Dorweiler JE, Carey CC, Kubo KM, Hollick JB, Kermicle JL, et al (2000) *mediator of paramutation1* is required for establishment and maintenance of paramutation at multiple maize loci. Plant Cell 12: 2101–2118. 11090212 10.1105/tpc.12.11.2101 PMC150161

[9] Alleman M, Sidorenko L, McGinnis K, Seshadri V, Dorweiler JE, et al (2006) An RNA-dependent RNA polymerase is required for paramutation in maize. Nature 442: 295–298. 16855589 10.1038/nature04884

[10] Erhard KF Jr, Stonaker JL, Parkinson SE, Lim JP, Hale CJ, et al (2009) RNA polymerase IV functions in paramutation in *Zea mays* . Science 323: 1201–1205. 19251626 10.1126/science.1164508

[11] Sidorenko L, Dorweiler JE, Cigan AM, Arteaga-Vazquez M, Vyas M, et al (2009) A dominant mutation in *mediator of paramutation2*, one of three second-largest subunits of a plant-specific RNA polymerase, disrupts multiple siRNA silencing processes. PLoS Genet 5: e1000725 doi:10.1371/journal.pgen.1000725. 19936058 10.1371/journal.pgen.1000725 PMC2774164

[12] Stonaker JL, Lim JP, Erhard KF Jr, Hollick JB (2009) Diversity of Pol IV function is defined by mutations at the maize *rmr7* locus. PLoS Genet 5: e1000706 doi:10.1371/journal.pgen.1000706. 19936246 10.1371/journal.pgen.1000706 PMC2775721

[13] Pikaard CS, Haag JR, Ream T, Wierzbicki AT (2008) Roles of RNA polymerase IV in gene silencing. Trends in Plant Science 13: 390–397. 18514566 10.1016/j.tplants.2008.04.008 PMC2679257

[14] Herr AJ, Jensen MB, Dalmay T, Baulcombe DC (2005) RNA polymerase IV directs silencing of endogenous DNA. Science 308: 118–120. 15692015 10.1126/science.1106910

[15] Onodera Y, Haag JR, Ream T, Costa Nunes P, Pontes O, et al (2005) Plant nuclear RNA polymerase IV mediates siRNA and DNA methylation-dependent heterochromatin formation. Cell 120: 613–622. 15766525 10.1016/j.cell.2005.02.007

[16] Brink RA, Styles ED (1966) A collection of pericarp factors. Maize Genetics Cooperation Newsletter 40: 149–160.

[17] Chopra S, Athma P, Peterson T (1996) Alleles of the maize P gene with distinct tissue specificities encode Myb-homologous proteins with C-terminal replacements. Plant Cell 8: 1149–1158. 8768374 10.1105/tpc.8.7.1149 PMC161193

[18] Chopra S, Athma P, Li XG, Peterson T (1998) A maize Myb homolog is encoded by a multicopy gene complex. Molecular and General Genetics 260: 372–380. 9870702 10.1007/s004380050906

[19] Das OP, Messing J (1994) Variegated phenotype and developmental methylation changes of a maize allele originating from epimutation. Genetics 136: 1121–1141. 8005419 10.1093/genetics/136.3.1121 PMC1205868

[20] Sekhon RS, Peterson T, Chopra S (2007) Epigenetic modifications of distinct sequences of the *p1* regulatory gene specify tissue-specific expression patterns in maize. Genetics 175: 1059–1070. 17179091 10.1534/genetics.106.066134 PMC1840062

[21] Cocciolone SM, Chopra S, Flint-Garcia SA, McMullen MD, Peterson T (2001) Tissue-specific patterns of a maize Myb transcription factor are epigenetically regulated. Plant Journal 27: 467–478. 11576430 10.1046/j.1365-313x.2001.01124.x

[22] Sidorenko LV, Li X, Cocciolone SM, Chopra S, Tagliani L, et al (2000) Complex structure of a maize *Myb* gene promoter: functional analysis in transgenic plants. Plant Journal 22: 471–482. 10886767 10.1046/j.1365-313x.2000.00750.x

[23] Cocciolone SM, Sidorenko LV, Chopra S, Dixon PM, Peterson T (2000) Hierarchical patterns of transgene expression indicate involvement of developmental mechanisms in the regulation of the maize *P1-rr* promoter. Genetics 156: 839–846. 11014829 10.1093/genetics/156.2.839 PMC1461292

[24] Chopra S, Cocciolone SM, Bushman S, Sangar V, McMullen MD, et al (2003) The maize *Unstable factor for orange1* is a dominant epigenetic modifier of a tissue specifically silent allele of *pericarp color1* . Genetics 163: 1135–1146. 12663550 10.1093/genetics/163.3.1135 PMC1462483

[25] Sekhon RS, Chopra S (2009) Progressive loss of DNA methylation releases epigenetic gene silencing from a tandemly repeated maize *Myb* gene. Genetics 181: 81–91. 19001287 10.1534/genetics.108.097170 PMC2621191

[26] Sidorenko LV, Peterson T (2001) Transgene-induced silencing identifies sequences involved in the establishment of paramutation of the maize *p1* gene. Plant Cell 13: 319–335. 11226188 10.1105/tpc.13.2.319 PMC102245

[27] Sidorenko L, Chandler V (2008) RNA-dependent RNA polymerase Is required for enhancer-mediated transcriptional silencing associated with paramutation at the maize *p1* gene. Genetics 180: 1983–1993. 18845841 10.1534/genetics.108.095281 PMC2600936

[28] Sidorenko L, Li X, Tagliani L, Bowen B, Peterson T (1999) Characterization of the regulatory elements of the maize *P-rr* gene by transient expression assays. Plant Molecular Biology 39: 11–19. 10080705 10.1023/a:1006172815663

[29] Lund G, Messing J, Viotti A (1995) Endosperm-specific demethylation and activation of specific alleles of alpha-tubulin genes of Zea mays L. Mol Gen Genet 246: 716–722. 7898439 10.1007/BF00290717

[30] Haring M, Bader R, Louwers M, Schwabe A, van Driel R, et al (2010) The role of DNA methylation, nucleosome occupancy and histone modifications in paramutation. The Plant Journal 63: 366–378. 20444233 10.1111/j.1365-313X.2010.04245.x

[31] Johnson LM, Bostick M, Zhang X, Kraft E, Henderson I, et al (2007) The SRA methyl-cytosine-binding domain links DNA and histone methylation. Current Biology 17: 379–384. 17239600 10.1016/j.cub.2007.01.009 PMC1850948

[32] Bernatavichute YV, Zhang X, Cokus S, Pellegrini M, Jacobsen SE (2008) Genome-wide association of histone H3 lysine nine methylation with CHG DNA methylation in *Arabidopsis thaliana* . PLoS ONE 3: e3156 doi:10.1371/journal.pone.0003156. 18776934 10.1371/journal.pone.0003156 PMC2522283

[33] Estève P-O, Chin HG, Smallwood A, Feehery GR, Gangisetty O, et al (2006) Direct interaction between DNMT1 and G9a coordinates DNA and histone methylation during replication. Genes and Development 20: 3089–3103. 17085482 10.1101/gad.1463706 PMC1635145

[34] Malagnac F, Bartee L, Bender J (2002) An Arabidopsis SET domain protein required for maintenance but not establishment of DNA methylation. EMBO Journal 21: 6842–6852. 12486005 10.1093/emboj/cdf687 PMC139107

[35] Jackson JP, Lindroth AM, Cao X, Jacobsen SE (2002) Control of CpNpG DNA methylation by the KRYPTONITE histone H3 methyltransferase. Nature 416: 556–560. 11898023 10.1038/nature731

[36] Grotewold E, Athma P, Peterson T (1991) Alternatively spliced products of the maize *P* gene encode proteins with homology to the DNA-binding domain of *myb*-like transcription factors. Proceedings of the National Academy of Sciences of the United States of America 88: 4587–4591. 2052542 10.1073/pnas.88.11.4587 PMC51710

[37] Saghai-Maroof MA, Soliman KM, Jorgensen RA, Allard RW (1984) Ribosomal DNA spacer-length polymorphisms in barley: Mendelian inheritance, chromosomal location, and population dynamics. Proceedings of the National Academy of Sciences of the United States of America 81: 8014–8018. 6096873 10.1073/pnas.81.24.8014 PMC392284

[38] Lechelt C, Peterson T, Laird A, Chen J, Dellaporta SL, et al (1989) Isolation and molecular analysis of the maize *P* locus. Molecular and General Genetics 219: 225–234. 2559311 10.1007/BF00261181

[39] Jacobsen SE, Sakai H, Finnegan EJ, Cao X, Meyerowitz EM (2000) Ectopic hypermethylation of flower-specific genes in Arabidopsis. Current Biology 10: 179–186. 10704409 10.1016/s0960-9822(00)00324-9

[40] Haring M, Offermann S, Danker T, Horst I, Peterhansel C, et al (2007) Chromatin immunoprecipitation: optimization, quantitative analysis and data normalization. Plant Methods 3: 11. 17892552 10.1186/1746-4811-3-11 PMC2077865

[41] Kimura H, Hayashi-Takanaka Y, Goto Y, Takizawa N, Nozaki N (2008) The organization of histone H3 modifications as revealed by a panel of specific monoclonal antibodies. Cell structure and function 33: 61–73. 18227620 10.1247/csf.07035

