# Supplementary figures and images for: Maize Unstable factor for orange1 Is Required for Maintaining Silencing Associated with Paramutation at the pericarp color1 and booster1 Loci
Source: PLoS Genet. 2012 Oct 4;8(10):e1002980. doi: 10.1371/journal.pgen.1002980 (PMC3464198; doi:10.1371/journal.pgen.1002980)

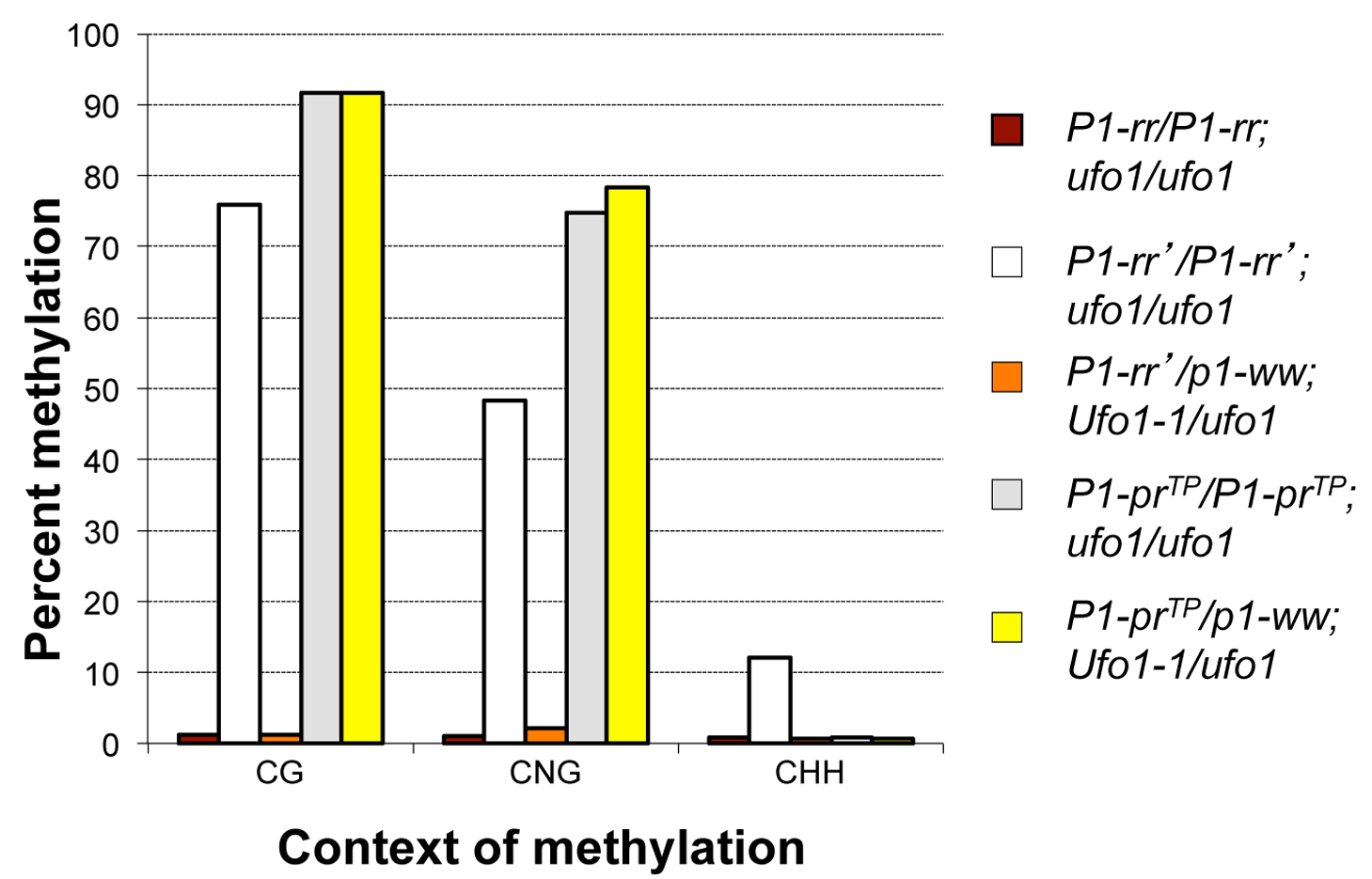

Supplement: Figure S1 — Comparison of overall cytosine methylation in the 443 bp fragment of the P1.2 enhancer region as assayed by genomic bisulfite sequencing. For each genotype, overall methylation in each context was calculated by dividing the number of methylated cytosines by the total number of cytosines in the context in all the clones. Context of methylation is on the x-axis and percentage of methylation is on the y-axis. (TIF) [file pgen.1002980.s001.tif]

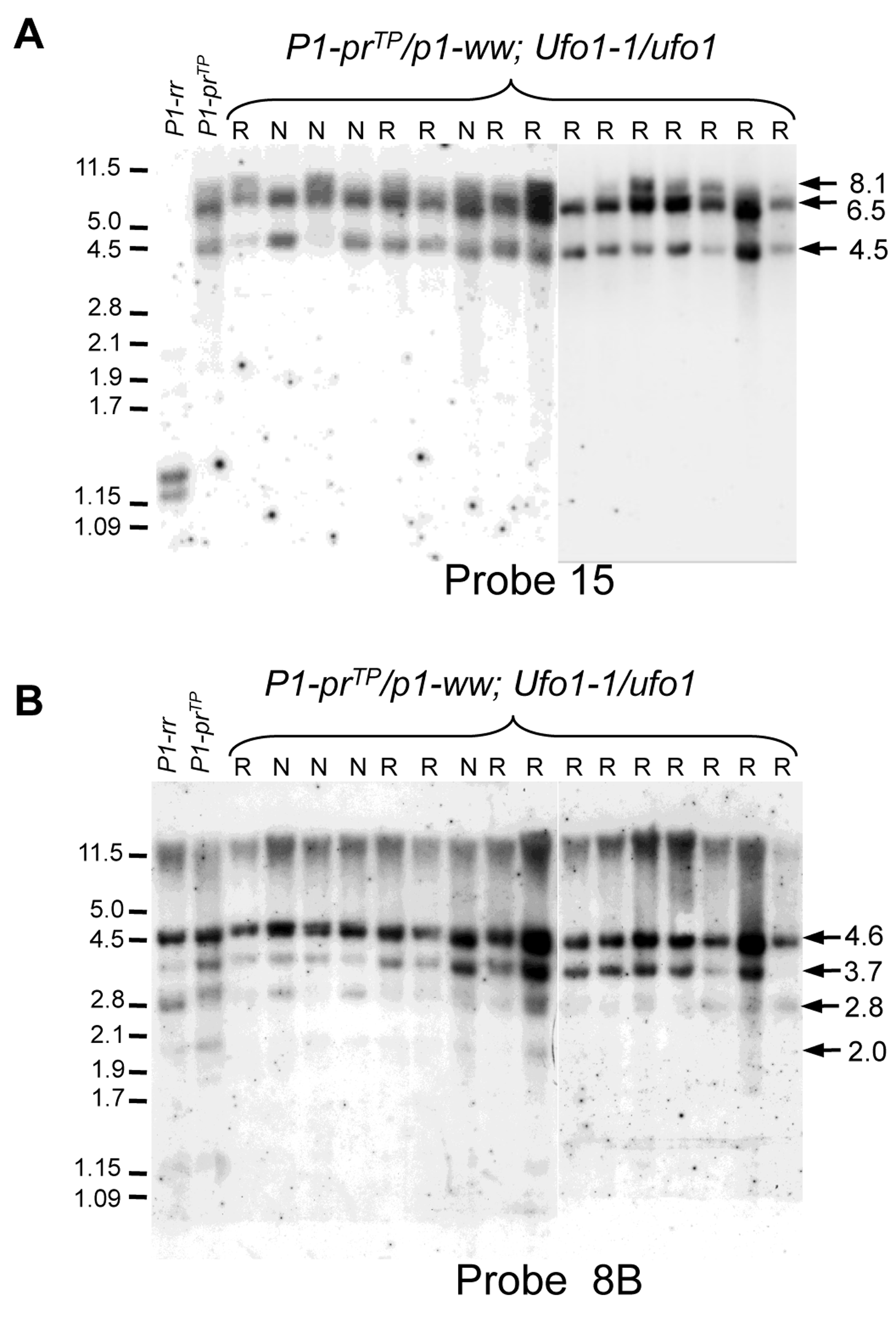

Supplement: Figure S2 — Effect of Ufo1-1 on methylation of P1-prTP. Gel blots carrying HpaII-digested leaf genomic DNA of P1-rr, P1-prTP, and P1-prTP Ufo1-1 plants with varying levels of pericarp pigmentation were hybridized with p1 probes; fragment 15 (A) and 8B (B). Molecular weights (in kilobases) of DNA ladder is shown on the left and sizes of hybridizing bands are shown on the right. Letters on the top of each lane indicate pericarp pigmentation of P1-prTP/p1-ww; Ufo1-1/ufo1: R for up regulated phenotype with pigmented pericarp; N for non-pigmented pericarps. (TIF) [file pgen.1002980.s002.tif]

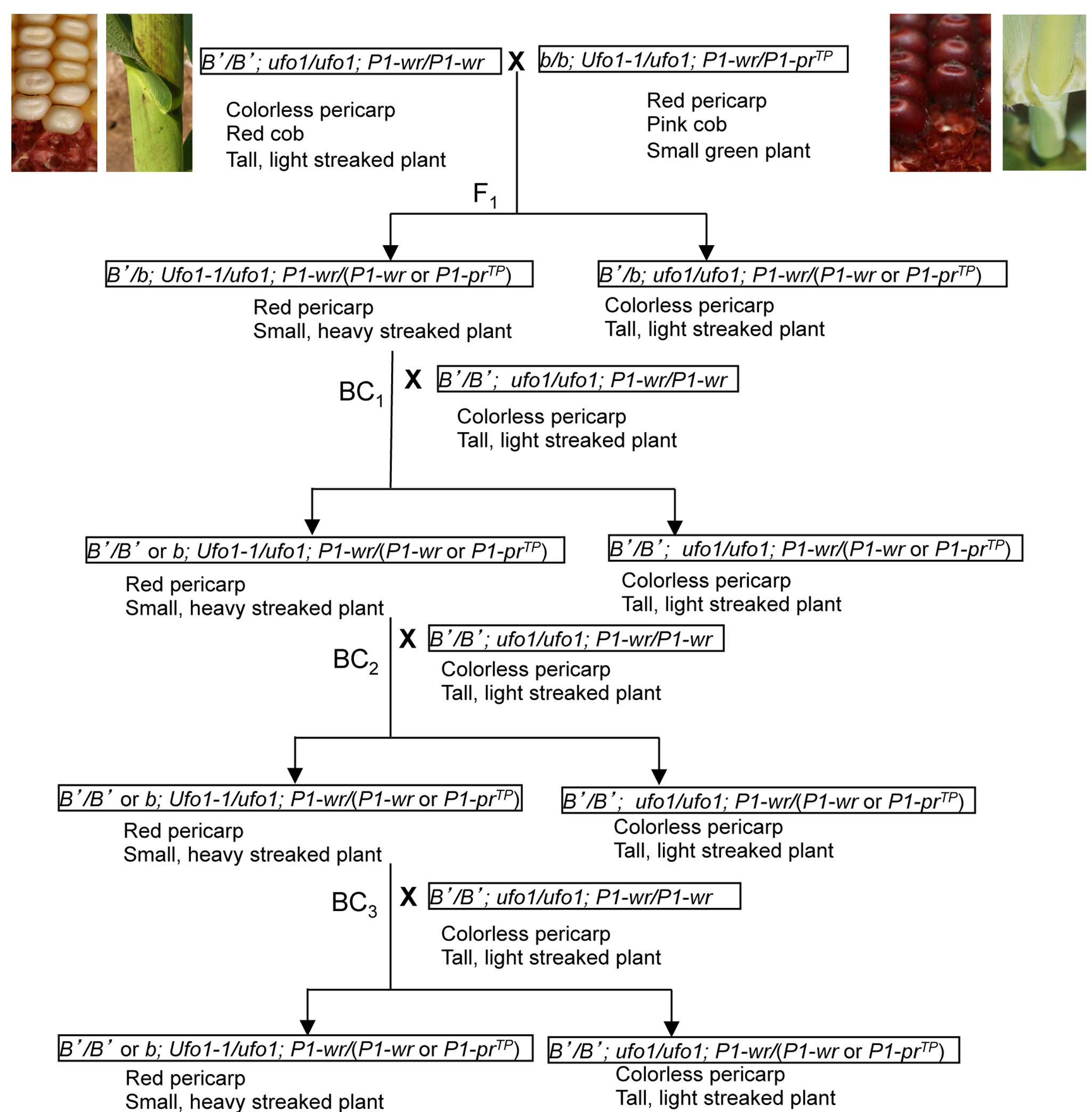

Supplement: Figure S3 — Crossing scheme used to test effect of Ufo1-1/ufo1 on B′. Homozygous B′ plants were crossed with the Ufo1-1/ufo1 stock which carried the neutral to paramutation b allele of the b1 gene. In the presence of Ufo1-1/ufo1, the P1-wr or P1-prTP plants display increased pericarp and cob glume pigmentation which was used to identify the Ufo1-1/ufo1 plants in segregating families. In cases when a Ufo1-1/ufo1 plant did not develop ear or ears failed to set seed, Ufo1-1/ufo1 plants were identified using a combination of phenotypic traits such as reddish/orange plant pigment, small stature, and characteristically bent tassel. To ensure that Ufo1-1 was passed on to the next generation, only ears with dark red pericarp pigmentation were used for planting. Because Ufo1-1 homozygotes did not survive in the hot and dry Arizona summer weather, experiment was carried out by repeated backcrossing Ufo1-1/ufo1 by the B′ ufo1 stock. Phenotypes of the up regulated B′ plants and data for all four generations are shown in Figure 8. (TIF) [file pgen.1002980.s003.tif]
